# Prevalence of eye and adnexal disorders among elderly inmates in Taiwan prisons

**DOI:** 10.1186/s12889-024-17796-4

**Published:** 2024-01-31

**Authors:** Ching-Yao Tsai, Zhu Liduzi Jiesisibieke, Ping Tao, Yen-Chun Wang, Dina Jiesisibieke, Ching-Wen Chien, Tao-Hsin Tung

**Affiliations:** 1 Department of Ophthalmology, Taipei City Hospital, Taipei, Taiwan; 2 Institute of Public Health, National Yang Ming Chiao Tung University, Taipei, Taiwan; 3 Department of Business Administration, Fu Jen Catholic University, Taipei, Taiwan; 4 General Education Center, University of Taipei, Taipei, Taiwan; 5 Evidence-based Medicine Center, Taizhou Hospital of Zhejiang Province affiliated to Wenzhou Medical University, Linhai, Zhejiang 317000 China; 6 School of Public Health, The University of Hong Kong Li Ka Shing Faculty of Medicine, Hong Kong, China; 7 Department of Medical Affairs and Planning, Section of Medical Fees, Kaohsiung Veterans General Hospital, Kaohsiung City, Taiwan; 8 Department of Public Health, College of Health Science, Kaohsiung Medical University, Kaohsiung, Taiwan; 9 Peking University Third Hospital, Beijing, China; 10 Institute for Hospital Management, Tsing Hua University, Shenzhen Campus, Shenzhen, China

**Keywords:** Elderly prisoners, Prevalence, Taiwan, Population-based

## Abstract

**Background:**

Prisoner health is a topic of significant importance; however, it has received limited attention in epidemiological studies, likely because of challenges in obtaining relevant data. Specifically, research on ocular disorders among elderly prisoners is lacking. Thus, the aim of this study was to evaluate the prevalence of disorders of the eye and adnexa among elderly prisoners in Taiwan.

**Methods:**

We investigated the presence of eye and adnexal disorders in elderly prisoners in Taiwan using data from the Taiwan National Health Insurance Research Database. The ocular disorders were identified using the appropriate disease codes in the International Classification of Diseases, 9th revision Clinical Modification (codes 360–379). In addition, the most common types of eye and adnexal disorders among the prisoners were identified.

**Results:**

A total of 2215 elderly prisoners (age ≥ 65 years; 2073 men and 142 women) were examined. The prevalence of eye and adnexal disorders among the prisoners was 18.87%. The elderly female prisoners exhibited a higher prevalence of eye and adnexal disorders than the elderly male prisoners. The most common disorders were disorders of the conjunctiva, cataract, and disorders of the lacrimal system.

**Conclusions:**

A considerable proportion of elderly prisoners have disorders of the eye and adnexa. The overall quality of life of elderly prisoners can be improved by addressing their visual health, which contributes to the fulfillment of their basic human rights.

## Introduction

Prisoner health is an important component of public health; however, prisoners often lack equal access to healthcare services because of the separation of prison health services from health services for the general public [1]. In addition, overcrowding, which can impede healthcare delivery, is a common problem in prisons, with nearly 70% of prisons affected [2]. Communicable diseases in prisons have been extensively studied; however, non-communicable diseases in prisons, particularly in low- and middle-income countries/regions, remain largely unexplored [3]. In Taiwan, comprehensive datasets on the health of prisoners, including their mental health [4] and skin health [5], are available. However, studies conducted using these datasets were primarily focused on broader prison populations and lack specific evidence regarding elderly prisoners.

Neglect and abuse of elderly prison inmates is a pressing issue [6]. Research suggests that the health status of elderly male prisoners is often worse than that of younger prisoners and the general public [7]. Elderly individuals, including prison inmates, are susceptible to age-related deterioration of visual health, which is significantly correlated with mental health [8, 9]. In addition, older age, low education level, and chronic diseases have been identified as risk factors for visual impairment in individuals with a history of criminal justice involvement [10]. Poor vision greatly affects quality of life, hindering everyday activities such as reading, exercising, and social engagement [9, 11]. The challenges that elderly prisoners face in maintaining good vision are exacerbated by their limited access to healthcare services and the suboptimal prison environments. Considering that more than 95% of prisoners will eventually reintegrate into society [12], it is crucial to address their health issues, which impose a burden on community health, particularly in impoverished communities.

The aim of this study was to determine the prevalence of disorders of the eye and adnexa among elderly prisoners in Taiwan. By examining the health status of this population, we can provide a comprehensive overview of the status of prisoner health in Taiwan, which will enable healthcare professionals identify and address health issues among prisoners and inform future health policy decisions.

## Methods

### Study design

This study was conducted to determine the prevalence of eye and adnexal disorders among elderly prisoners in Taiwan. Data used for this study were extracted from the Taiwan National Health Insurance Research Database (NHIRD), a large dataset that provides information on eye and adnexal disorders among individuals in Taiwan, including prisoners [13, 14]. The NHIRD was established to regulate health data following the implementation of the mandatory National Health Insurance program in 1995 [14]. It includes three forms of data: a general dataset, a disease-specific dataset, and a full population dataset [15]. The NHIRD has been used extensively by researchers for the collation of epidemiological information on healthcare in Taiwan [16, 17]. The International Classification of Diseases, 9th Revision Clinical Modification (ICD-9-CM), is used for coding diseases in the dataset. Previous studies have provided in-depth information on the NHIRD, offering researchers a comprehensive understanding of its capabilities [4, 5]. In this research, we identified inmates from a specific data set encompassing all inmates from January 1, 2013 to December 31, 2013 in the NHIRD. The research ethics of conducting studies using NHIRD present significant challenges and indicate the need for formal review processes [4]. The ethical frameworks for NHIRD research are subject to intense debate and are constantly evolving, given the uncertain potential risks associated with data science research [18]. This study was approved by the Institutional Review Board of Cheng-Hsin General Hospital (CHGH-IRB: [471]104-07). All patient data were handled in accordance with the guidelines of the Declaration of Helsinki guidelines to ensure patient anonymity.

### Study population

This study specifically focused on individuals aged 65 years or older. Disorders of the eye and adnexa were identified using the ICD-9 codes 360–379. The prevalence of different subtypes of eye and adnexal among the inmates was analyzed as well.

### Statistical analysis

Statistical analyses were performed using SAS version 9.1 developed by SAS Institute Inc. (Cary, NC, USA). Categorical variables and prevalence are presented as absolute frequencies and percentages. The χ^2^ method was used to evaluate the significance of differences in the proportions of categorical variables in the univariate analysis. Statistical significance was set at *p* < 0.05.

## Results

A total of 2,215 prisoners (men, *n* = 2073; women, *n* = 142) were analyzed in this study (Table 1). The average age of the female prisoners was 70.00 years, whereas that of the male prisoners was 69.83 years. The average age of the female prisoners with eye and adnexal disorders was 70.62 years, whereas that of the males was 70.88 years. The age distribution patterns observed in the entire population of elderly prisoners and the population of prisoners with eye and adnexal disorders were similar. Notably, the elderly prisoners with eye and adnexal disorders required medical services at a rate 1.47 times higher than that for the inmates in all.

**Table 1:** Demographic data of the study population categorized according to sex (Taiwan, 2013)

	Total		Disorders of the eye and adnexa	
	Female (*n* = 142)	Male (*n* = 2073)	Female (*n* = 37)	Male (*n* = 381)
Age				
Mean (standard deviation)	70.00 (5.18)	69.83 (5.37)	70.62 (5.22)	70.88 (5.46)
Range (min-max)	65–88	65–103	65–88	65–96
Utilization of medical services (number per year)				
Mean (standard deviation)	30.79 (18.15)	24.80 (19.06)	36.11 (19.62)	37.06 (24.13)
Range (min-max)	2-122	1-165	15–122	3-165

The prevalence of disorders of the eye and adnexa among the elderly prisoners was 18.87%. The most common disorders of the eye and adnexa diagnosed in the study population were disorders of the conjunctiva, which accounted for 12.10% of all cases, followed by cataract (ICD9_366, 7.72%) and disorders of the lacrimal system (ICD9_375, 2.62%) (Table 2). Men showed a lower prevalence of eye and adnexal disorders than women (*p* = 0.0237). There was a noticeable disparity between the prevalence of conjunctival disorders and that of disorders of the lacrimal system.

**Table 2:** Prevalence of the most common eye and adnexal disorders among the prisoners (*N* = 2215, Taiwan, 2013)

	Total	Female		Male		P for X2 test
	%	*n*	%	*n*	%	
Total number of prisoners		142	6.41	2073	93.59	
ICD9_360–379 Disorders of the eye and adnexa	18.87	37	26.06	381	18.38	0.0237
ICD9_366- Cataract	7.72	14	9.86	157	7.57	0.3236
ICD9_368- Visual disturbances	2.17	6	4.23	42	2.03	0.0521
ICD9_370- Keratitis	1.04	2	1.41	21	1.01	0.2602
ICD9_372- Disorders of the conjunctiva	12.10	27	19.01	241	11.63	0.0090
ICD9_373- Inflammation of the eyelids	1.08	3	2.11	21	1.01	0.1329
ICD9_375- Disorders of the lacrimal system	2.62	8	5.63	50	2.41	0.0191

The sex-specific prevalence of disorders of the eye and adnexa are shown in Table 3. Regarding the elderly female prisoners, 26.06% had eye and adnexal disorders, with conjunctival disorders (ICD9_372) being the most frequently diagnosed (19.01%), followed by cataract (ICD9_366, 9.86%), disorders of lacrimal system (ICD9_375, 5.63%), and visual disturbances (ICD9_368, 4.23%). For the elderly male prisoners, 18.38% of them had disorders of the eye and adnexa, with the most commonly diagnosed disorders being conjunctival disorders (ICD9_372, 11.63%), cataract (ICD9_366, 7.57%), and other retinal disorders (ICD9_362, 2.51%).

**Table 3:** Sex-specific prevalence of eye and adnexal disorders among the prisoners (*N* = 2215; Taiwan, 2013)

	Female			Male		
	*n*	%	Mean age (SD)	*n*	%	Mean age (SD)
**Total number of prisoners**	142	6.41	70.00(5.18)	2073	93.59	69.83(5.37)
**ICD9_360–379 Disorders of the eye and adnexa**	37	26.06	70.62(5.22)	381	18.38	70.88(5.46)
ICD9_360- Disorders of the globe	1	0.70	-	3	0.14	75.33(2.52)
ICD9_361- Retinal detachments and defects	0		-	4	0.19	66.00(0.82)
ICD9_362- Other retinal disorders	3	2.11	73.33(10.97)	52	2.51	71.15(5.58)
ICD9_363- Chorioretinal inflammations and scars and other disorders of the choroid	0		-	1	0.05	-
ICD9_364- Disorders of the iris and ciliary body	0		-	2	0.10	71.50(9.19)
ICD9_365- Glaucoma	4	2.82	70.50(3.70)	36	1.74	70.39(5.49)
ICD9_366- Cataract	14	9.86	73.14(6.78)	157	7.57	71.44(5.51)
ICD9_367- Disorders of refraction and accommodation	3	2.11	66.67(0.58)	20	0.96	70.80(3.30)
ICD9_368- Visual disturbances	6	4.23	72.00(3.46)	42	2.03	71.14(4.98)
ICD9_369- Blindness and low vision	0		-	2	0.10	72.00(4.24)
ICD9_370- Keratitis	2	1.41	73.00(2.83)	21	1.01	71.00(4.77)
ICD9_371- Corneal opacity and other disorders of the cornea	0		-	7	0.34	74.57(6.70)
ICD9_372- Disorders of the conjunctiva	27	19.01	70.70(4.63)	241	11.63	70.85(5.15)
ICD9_373- Inflammation of the eyelids	3	2.11	67.67(3.79)	21	1.01	70.38(5.06)
ICD9_374- Other disorders of the eyelids	0		-	17	0.82	74.12(5.43)
ICD9_375- Disorders of the lacrimal system	8	5.63	69.88(2.59)	50	2.41	70.78(4.84)
ICD9_376- Disorders of the orbit	0		-	2	0.10	68.50(4.95)
ICD9_377- Disorders of the optic nerve and visual pathways	0		-	2	0.10	69.00(5.66)
ICD9_378- Strabismus and other disorders of binocular eye movements	0		-	1	0.05	-
ICD9_379- Other disorders of the eye	1	0.70	-	22	1.06	71.27(7.24)

## Discussion

Previous studies have shown that prisoners in Taiwan have a worse health status than the general public [4, 5]. This study indicated that the prevalence of eye and adnexal disorders among elderly prisoners in Taiwan is 18.87%, which is relatively high [19]. Further analysis of the sex-specific differences in visual health among prisoners revealed that the prevalence of eye and adnexa disorders among the female elderly prisoners is higher than that among the male elderly prisoners. Conjunctival disorders were the most common eye disorders observed in the study population.

An observational study indicated that the rates of blindness and low vision in Taiwan are 0.59% and 2.94%, respectively among elderly Chinese population in Taiwan [19]. The prevalence of eye and adnexal disorders among the elderly prisoners analyzed in the present study was 18.87%. Similar studies conducted in prisons in other countries indicated a high prevalence of eye disorders among prison inmates as well. In Nigerian prisons, 26.8% of the inmates have disorders of the eye and adnexa [20]. Another study conducted in Italy indicated that prisoners reported more eye problems while serving time [21]. Moreover, limited access to healthcare services, including early screening, prevention, and treatment, may aggravate this problem.

We analyzed sex-specific differences in the prevalence of eye and adnexal disorders in the study population. The results showed that the prevalence of eye and adnexal disorders among the female elderly prisoners was higher than that among the male elderly prisoners. Sex-specific differences in neuro-ophthalmological disorders, hereditary ocular disorders, and age-related macular degeneration have been analyzed in previous studies. In addition, several previous studies have been conducted to determine whether the effect of sex hormones on ocular blood flow and neural function is the reason underlying the sex-specific differences in the prevalence of ocular disorders [22]. However, all these studies were conducted using participants from the general population, rather than prison inmates. Thus, further research conducted using populations of prison inmates is needed to clarify and address the sex-specific differences in health issues specific to prisons. Furthermore, it is important to consider the potential influence of security measures and limited resources on the accessibility of medical care for inmates, especially male prisoners [23]. Previous study also suggests that male inmates may exhibit a lower propensity to seek medical assistance in comparison to their female counterparts [24].

The top three most common ocular disorders observed in the study population were conjunctival disorders, cataract, and disorders of the lacrimal system. These disorders are different from the most common ocular disorders in the general population of Taiwan. The leading cause of visual impairment in the general Taiwan population is cataracts (41.7%), followed by myopic macular degeneration (12.5%) and age-related macular degeneration (10.4%) [19]. The higher prevalence of conjunctival disorders (especially infective conjunctivitis) among the inmates may be related to the living conditions in prisons, such as overcrowding [25].

Prisoner health is an important aspect of public health. It is estimated that inmates seek medical care in prison three to four times more frequently than the general public [26]. Healthcare delivery in prisons can be costly and unsafe because the inmates often need to be transported to medical centers for comprehensive examination and treatment. Several methods and interventions, such as early screening, early treatment, balanced nutrition, reduction of overcrowding, and use of remote diagnostic systems and networks, can help decrease the occurrence and transmission of diseases [27–30].

This study has several strengths. First, we utilized data extracted from the NHIRD in Taiwan, which included the medical information of all elderly inmates in prisons across the region, thus ensuring comprehensive representation of the elderly prisoner population. Second, the present study provides valuable epidemiological data on the prevalence of ocular and adnexal disorders among elderly prisoners, which can inform the development of policies for the improvement of prisoner health and welfare. Although epidemiological studies receive considerable attention because they are typically focused on the associations between risk factors and specific diseases, descriptive epidemiological studies, particularly those focusing on specific groups, have been largely overlooked. Specifically, descriptive research on the prevalence of ocular and adnexal disorders among elderly prisoners in Taiwan is limited. Our study addresses this knowledge gap and emphasizes the importance of preventing and treating ocular and adnexal disorders among elderly prison inmates.

This study has some limitations as well. First, given that we only included prisoners in Taiwan, the generalizability of our findings to other regions may be limited. Nevertheless, our research may still inspire policymakers and prison staff to prioritize the health statuses of elderly prisoners. Second, although the findings of this descriptive study indicate a high prevalence of disorders of the eye and adnexa among elderly prisoners in Taiwan, the associated factors for these disorders were not analyzed. Subsequent studies should focus on identifying these associated factors in order to develop more effective prevention strategies for ocular and adnexal disorders among prison inmates. Additionally, it is worth noting that the NHIRD does not provide information on socioeconomic status and biochemical factors. Therefore, it would be valuable to investigate these factors in future studies using alternative datasets. Third, it is important to recognize that this study is cross-sectional in nature. As a result, the prevalence of eye and adnexal disorders is solely based on diagnosis records from 2013, which may introduce some bias due to the lack of prior records. Fourth, due to limitations in data access, we were only able to obtain medical information for the prisoners in 2013, therefore, it is very difficult to compare the prevalence of eye and adnexal disorders between elderly prisoners and general elderly population in Taiwan. Fifth, the significance of χ^2^ tests is heavily reliant on the size of the sample. Consequently, even minor discrepancies in small sample sizes can yield statistical significance [31, 32]. Finally, it is important to note that the results of certain clinical tests were unavailable for analysis.

## Conclusions

The study revealed that a significant proportion of elderly prisoners have ocular and adnexal disorders. Furthermore, this study found that the most prevalent disorders of the eye and adnexa in this population were conjunctival disorders. Notably, the female elderly inmates showed a higher prevalence of eye and adnexal disorders than the male elderly inmates. Considering these findings, it may be beneficial to implement specialized intervention strategies specifically targeted at improving the visual health of female elderly prisoners. Enhancing visual health can improve the overall quality of life of elderly prisoners and contribute to the fulfillment of their fundamental human rights.

## Data Availability

All data underlying the findings are within the paper.

## Abbreviations

NHIRD: National Health Insurance Research Database
ICD-9-CM: International Classification of Diseases,9th Revision Clinical Modification
